# Individual Escape Flight Performance Is Repeatable and Differs Between Long‐Tailed Finch (*Poephila acuticauda*) Subspecies but Not as Predicted by Asymmetrical Introgression

**DOI:** 10.1002/ece3.73287

**Published:** 2026-03-30

**Authors:** Samuel Ashby, Callum S. McDiarmid, Dylan Dooner, Simon C. Griffith, Wiebke Schuett

**Affiliations:** ^1^ School of Life Sciences University of Sussex Falmer Brighton UK; ^2^ School of Natural Sciences Macquarie University Sydney New South Wales Australia; ^3^ School of Aerospace, Mechanical, and Mechatronic Engineering University of Sydney Sydney New South Wales Australia

**Keywords:** 3D tracking, Argus, hybrid, individual variation, natural selection, take‐off performance

## Abstract

An emerging question in evolutionary research is the extent to which mitochondrial variation across species and populations drives functional differences in fitness‐related traits. The asymmetric introgression and narrow mitochondrial clines seen in long‐tailed finch ( *Poephila acuticauda* ) subspecies is suggestive of selection but there is no direct evidence of fitness related differences in admixed birds. Asymmetric introgression of the eastern *P. a. hecki* mito‐type into *P. a. acuticauda*, suggests that the former may possess a selective energetic advantage such as superior escape flight performance. We employed a spatial tracking software, Argus, to quantify the take‐off flight performance of 158 wild‐derived, captive long‐tailed finches as they were released repeatedly, from which we obtained nine flight metrics. Average force and maximum vertical distance travelled were repeatable across all individuals and significantly lower in *P. a. hecki* compared to *P. a. acuticauda* males. Hence, contrary to predictions, *P. a. hecki* did not display escape flight performance superior to *P. a. acuticauda* suggesting take‐off flight performance in wild long‐tailed finches is not related to the observed asymmetry in the introgression of mito‐type between the subspecies. Furthermore, in intraspecific female hybrids, in which we might theoretically expect a mismatch between mitochondrial haplotype and mitonuclear genes, we also found no evidence of impaired flight take‐off performance, relative to those with matched mitochondrial and mitonuclear genes (here: female *P. a. acuticauda*). Finally, male and female hybrids did not differ in the average force and maximum vertical distance travelled, again suggesting that take‐off performance is not significantly worse in the heterogametic sex, as predicted by Haldane's rule. The angle of the take‐off flight was a key determinant of our flight performance metrics and consequently, future work could benefit from better directing the flight path of tracked birds.

## Graphical abstract

We repeatedly tested 158 individual long‐tailed finches, *Poephila acuticauda*, of two different sub‐species and their hybrids for differences in their flight performances. Several flight performance variables were repeatable across individuals. Contrary to expectations, *P. a. hecki* individuals did not display superior flight performance to *P. a. acuticauda* individuals and hybrids showed no impaired performance.

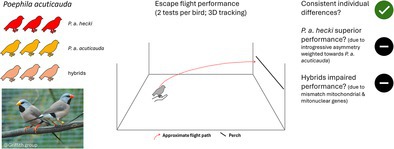

## 1 Introduction

When considering the isolating mechanisms that maintain or accelerate divergence between closely related species, the phenotype and performance of hybrids, relative to pure parental forms, can provide some insight into the mechanisms that reduce introgression of genes from one species into another when they are in contact (Price 2008). For example, moult schedules in hybrids can disrupt the timing of optimal migration (Rohwer and Irwin 2011; Rohwer et al. 2023); hybrids take intermediate or inferior migratory paths (Delmore and Irwin 2014); hybrids have significantly reduced cognitive abilities with respect to an associative learning task and a neophobia test (Alario et al. 2023; McQuillan et al. 2018); and hybrids have significantly different basal metabolic rate compared to parental forms (Tieleman et al. 2009; McFarlane et al. 2016). A relatively new framework suggests that divergence in both the mitochondrial genes and the nuclear genes that interact with the mitochondrial genes (mitonuclear genes) may underlie many differences in phenotype between closely divergent species, and the hybrids between them (Koch et al. 2021; Brand et al. 2024). In their study of the yellow‐rumped warbler (*Setophaga* [*coronata*] spp.) species complex, Toews et al. (2014) identified divergence in mitochondria between subspecies and a cryptic contact zone in which the mitochondrial DNA of one subspecies was introgressing into another (asymmetric introgression), whilst also finding that mitonuclear discordance was greatest in a non‐migratory subspecies, where selection on their capacity to produce ATP would not be as strong as in subspecies that migrate. This study by Toews et al. (2014) suggests that even closely related and inter‐breeding subspecies can differ functionally in their capacity to synthesise cellular energy.

Here we have examined two subspecies of long‐tailed finch ( *Poephila acuticauda acuticauda* and *P. acuticauda hecki*, hereafter *acuticauda* and *hecki*) that occupy northern Australia and are separated by relatively steep clines in nuclear genetic (Hooper et al. 2019), mitochondrial (Lopez et al. 2021) and bill colour divergence (Griffith and Hooper 2017; Hooper et al. 2024). The narrowness of the clines in genetic variation (mostly due to a Z chromosomal inversion; Hooper et al. 2019) (147 km) and mitochondrial variation (32 km) (Lopez et al. 2021) between the two subspecies suggests that there are appreciable costs to hybridisation of these forms. Furthermore, the three clines are geographically displaced from one another, with the nuclear genetic and mitochondrial clines being 334 and 394 km to the east of the bill colour cline, respectively (Lopez et al. 2021).

After accounting for the levels of divergence within each subspecies, the level of nucleotide divergence between *acuticauda* and *hecki* is 24 times higher for the mitochondrial genome compared with that found across the autosomes, and the functional performance of mitochondrial respiration are significantly different in the two subspecies when measured in the laboratory (Pacheco‐Fuentes et al. 2025). In this recent experimental study that measured mitochondrial function both before and after exposure to extreme heatwave conditions, the eastern subspecies *hecki* had a mitochondrial profile that provides greater protection from oxidative stress and higher metabolic flexibility compared to the western subspecies *acuticauda* (Pacheco‐Fuentes et al. 2025). These functional differences in the mitochondrial haplotypes of the two subspecies are consistent with the finding that the *hecki* mito‐type has introgressed further into the zone of the *acuticauda* mito‐type than vice‐versa (McDiarmid et al. 2024). The asymmetrical representation of parental genotypes on the two sides of a contact zone theoretically suggests that positive selection may be acting on some phenotypes (e.g., Johannesen et al. 2006; Jezkova et al. 2013; Metzler et al. 2021) although other factors such as genetic drift or epistatic interactions might also be responsible.

Variation in mitochondrial respiration between the subspecies might be reflected in their performance in energetically demanding activities such as flight (e.g., Toews et al. 2014), and here we might expect *hecki* to outperform *acuticauda*, given the asymmetry in the introgression of mitochondrial genes from east (*hecki*) to west (*acuticauda*) (Lopez et al. 2021). Alongside the predicted phenotypic differences between the two subspecies, there are also reasons to predict differences in the performance of F1 hybrids compared to parental forms. Given that nuclear divergence between the two subspecies is almost completely found on the Z chromosome (> 99.9% of fixed differences; Hooper et al. 2019), and the mitochondria is always matrilineally inherited we would expect F1 females to be more adversely affected than F1 males. That is because an F1 hybrid daughter will inherit the mitochondria from her mother, but the Z chromosome from her father, who will be from the other subspecies. As such, an F1 hybrid female will be mitodiscordant for mitonuclear genes on the Z chromosome. By contrast, an F1 hybrid male will also inherit the mitochondria from his mother, and one of his two Z chromosomes also from his mother. As such, his mitochondrial genes will come from the same genetic background as one of his Z chromosomes and will therefore at least partially match, i.e., he will be mitoconcordant. A recent study of these two subspecies has indeed identified mitochondria—nuclear incompatibilities affecting mitochondrial respiration of embryonic tissue in backcrossed individuals (McDiarmid et al. 2024). In particular, it was found that the mitodiscordance was greatest for embryos with an *acuticauda* mitochondria in a *hecki* background (McDiarmid et al. 2024), consistent with the observation that *hecki* mitochondrial genes are introgressing into *acuticauda* but not vice versa (Lopez et al. 2021).

Flight performance is fundamental for survival and reproduction in many bird species, facilitating predator evasion (e.g., Metcalfe and Ure 1995), dispersal and foraging (e.g., Marchetti et al. 1995) and courtship (e.g., Clark 2009). Therefore, flight performance is likely under selection, where individuals with impaired flight performance probably have decreased evolutionary fitness. Quantifying the efficiency of bird flight and its variation across individuals, populations, or species is therefore an area of research that has the capacity to provide insight into speciation processes through the lens of the mitonuclear compatibility species concept (Hill 2017).

Here, we aimed to use Argus to assess the flight performance of 158 individual long‐tailed finches repeatedly, including individuals of both subspecies and their first‐generation hybrids, to investigate whether there is evidence for divergence between the parental subspecies and whether mitodiscordance in F1 hybrids might be contributing to isolation of the two subspecies.

## 2 Methods

### 2.1 Study Species

The long‐tailed finches used in this study are part of the wild‐derived populations of both subspecies maintained at Macquarie University since 2010 (Hurley et al. 2018). Individuals from each of the two subspecies were included, as well as F1 hybrid individuals, i.e., individuals with one parent of each subspecies. This study used 158 finches in total, including 73 *acuticauda* (35 females, 38 males), 41 *hecki* (all male) and 44 F1 hybrids (19 females, 25 males) all of which had an *acuticauda* mother and a *hecki* father. We focused on these particular F1 hybrids given the previous finding in embryos that *acuticauda* mitochondria performed poorly in a *hecki* nuclear background (Lopez et al. 2021; McDiarmid et al. 2024), and to specifically test whether this holds at the level of an adult for a whole‐body performance task. All individuals were sexually mature and ranged from 1 to 6 years old. Seventy‐nine individuals (35 *acuticauda* females, 44 F1 hybrids) were housed in outdoor aviaries (0.95 [l] × 1.9 [w] × 1.8 m [h]), while 79 (41 *hecki* males, 38 *acuticauda* males) occupied indoor cages (0.8 [l] × 0.6 [w] × 0.47 m [h]). There were seven aviaries and six cages. Both enclosure types were installed with wooden perching platforms and birds were provisioned with a generic finch seed mix (*Panicum* and *Setaria* spp.), cuttlebone, grit and water ad libitum. Individuals were distinguished by coloured plastic leg bands with unique identification (ID) codes printed on them. Each individual was weighed [kg, precision ±0.1 g] prior to the study. Tarsometatarsus length ([mm]; hereafter, tarsus length) was measured, from the hallux to the tibiotarsus, for 98 individuals. For these 98 individuals, we calculated body condition as the residuals from a linear model of body mass (response variable) and tarsus length (explanatory variable).

### 2.2 Flight Trials

For the flight trials, all individuals within a given cage or aviary were collected into a holding box and taken to a separate empty elongated aviary (7.5 [l] × 1.7 [w] × 2.2 m [h]). We removed individuals one by one and wrote the corresponding ID upon a large display board captured by cameras. From one end of the aviary, the researcher would then release the bird towards the other end. Specifically, the researcher sat on the ground at one end of the aviary, held the bird in a standard ringer's grip in their right hand and placed the feet of the bird upon the top of the researcher's left hand, which was clenched and upon the ground. The bird was then released by suddenly opening the right hand. Released birds almost invariably flew towards a high perch at the far end of the aviary, which provided a safe refuge for them. Flight trials were repeated at least 6 days later, so that two flight trials were completed per individual.

The researcher performed various actions required by the tracking software Argus (see below); after the cameras were powered on and before they were powered off at the end of the recording session, the researcher clapped their hands three times, which facilitated later syncing of three videos (one per camera, see below). Before the first individual was released in each video, the researcher moved a 1‐m wand (a wooden rod with coloured balls at each end) in a ‘figure‐eight’ motion in front of the cameras, which facilitates distance calibration.

### 2.3 Flight Recording

**FIGURE 1:**
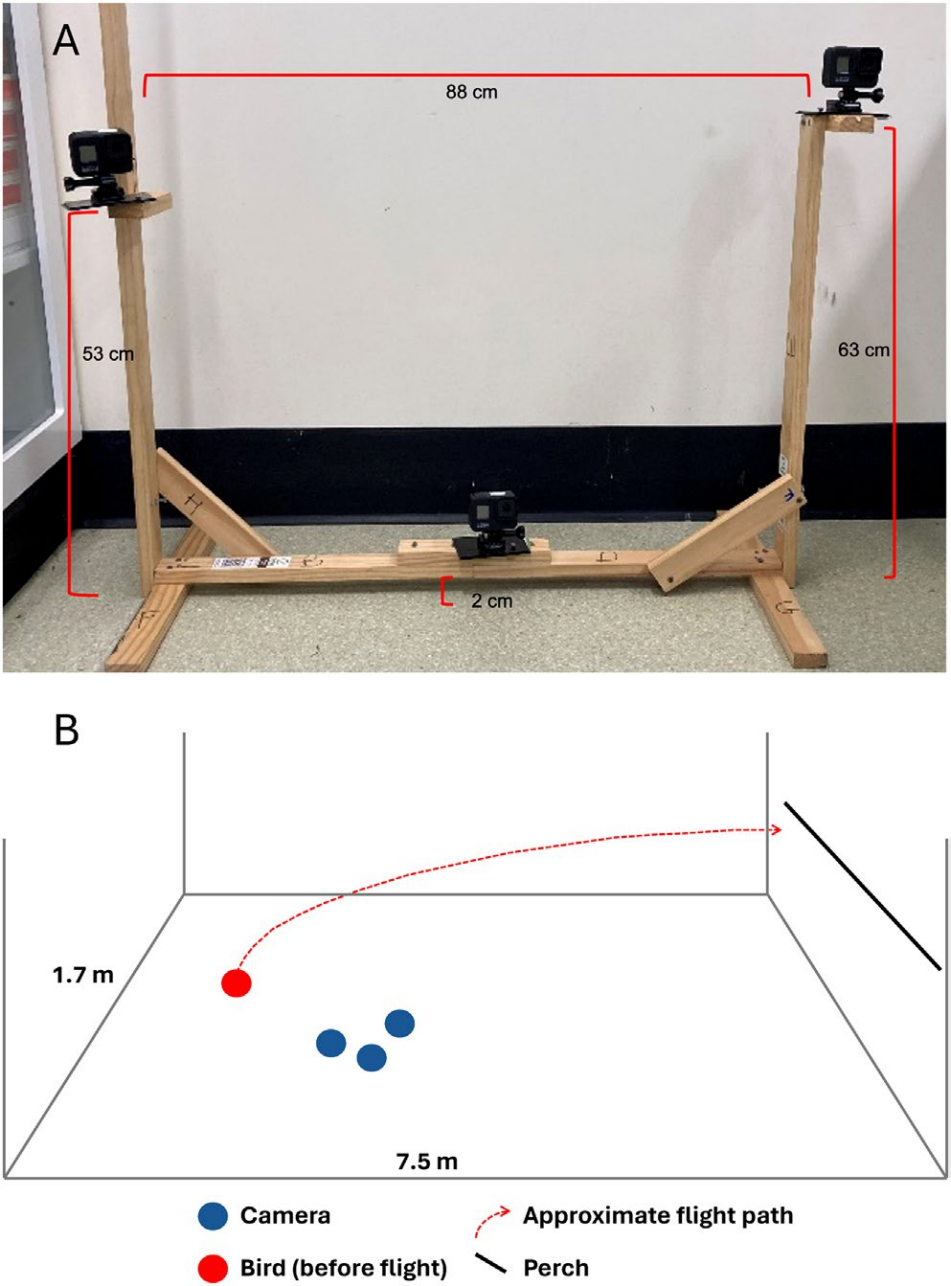
Flight setup. (A) Arrangement of GoPro Hero 8 cameras upon custom‐built apparatus. The triangular arrangement provided an enlarged field of view. (B) Arrangement of flight recording trials, where each bird is released (red circle) before flying towards a perch (black line). The cameras (blue) were positioned in an optimal position where the release and the flight path (red, dashed) could be recorded. Schematic not to scale.

Each flight was recorded by three GoPro Hero 8 (GoPro Inc., California, USA) cameras positioned in a triangular formation upon a wooden frame (Figure 1A) and angled towards the location of individual releases (Figure 1B). The positioning of the cameras was standardised across all flight trials. The cameras captured 240 frames per second (fps). Footage was written to secure digital (SD) cards in MP4 format. Each flight, therefore, was recorded in three videos, one per camera. The cameras recorded the entirety of a flight trial session, with the three videos forming one ‘video group’ per flight trial.

### 2.4 Flight Tracking

The flight footage was analysed via Argus (version 2.1; Jackson et al. 2016a; Jackson et al. 2016b), an open‐source program accessed through Python (version 3.6.2). The cameras were synchronised according to the occurrence of conspicuous sounds known as ‘beeps’, provided by the clapping before and after flight trials. The spatial parameters of the cameras were calibrated using the tracking of patterned dots (i.e., the camera angles were calibrated using video footage showing a paper with a grid of dots), following procedures outlined in the Argus documentation (Jackson et al. 2016b) and the movement of the one‐metre wand before tracking each individual's flight. In both instances, a specified point upon the wand or individual bird was clicked using the computer cursor. The wand was tracked for 500 frames per end of the wand, per flight trial session. The dark patch of black feathers around an individual's eye was used as a reference point to track each individual's flight. Once released, individuals were tracked for 150 frames. Nine flight tracks consisted of fewer than 150 frames, since the individual flew in an odd direction out of the area covered by the camera's field of view. These flight tracks were not included in the analyses. Finally, the output values obtained from Argus were transformed into coordinates of the tracked points into distance [m] travelled across the *x*, *y* and *z* axes. This process also provided three‐dimensional figures of each flight track. After processing and excluding failed trials, we had 291 flight tracks available for calculations of flight metrics and analysis.

**FIGURE 2:**
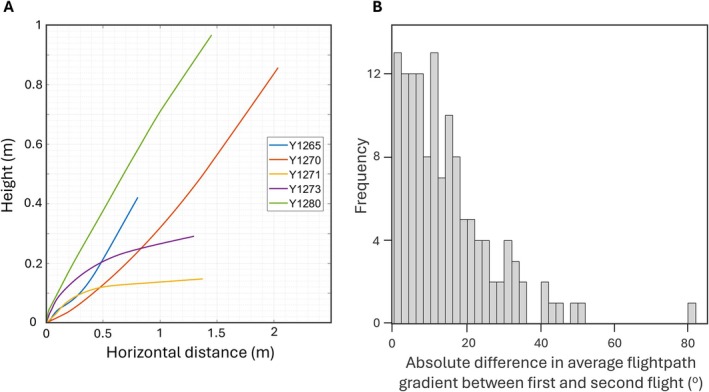
Long‐tailed finch flights. (A) Example of the smoothed flight paths of five long‐tailed finches during the 150 frames they were tracked. (B) Absolute difference in average flightpath gradient between the first and second flight of each long‐tailed finch. When calculating repeatability of the flight metrics, we explored limiting the dataset to only individuals with more minor differences in average flightpath gradient between their two flights (up to 20^o^ and up to 30^o^ difference, respectively).

MATLAB (version R2023b; The MathWorks Inc 2023) was used to calculate the flight metrics from the 3D tracks of the birds. The raw location data was first smoothed via linear least‐squares using a linear regression with a moving window internally determined by the black‐box smoothdata() function of MATLAB. The smoothed data was then zeroed so that every track started from zero on the *x*, *y* and *z* axes (Figure 2A). The metrics calculated were the total distance travelled [m], maximum horizontal distance [m], maximum vertical distance [m], average velocity [m/s], maximum velocity [m/s], average power [W], average force [N], the flightpath gradient (alpha [degrees]) averaged for the flight (i.e., the average angle off the ground the bird flew at) and the maximum flightpath gradient for the flight.

Specifically, the average force and average power were determined using the recorded time‐varying displacement data and individual bird masses. The maximum horizontal distance was calculated with the *x*‐ and *y*‐vector components of flight, with the maximum vertical distance as the maximum of the *z*‐flight vector. The total distance travelled is then the vector‐sum of these three components. From the total vertical distance gained, the energy potential gain can be determined, which is a function of the individual bird's work. Additionally, from these horizontal and vertical components, the flightpath gradient was computed at each time‐step. From this, the average and maximum flightpath gradient were determined by taking the average and maximum of the values across all time‐steps.

To determine the average power and force output by the birds, the flight velocity components were computed from the displacement data. This was performed using a second‐order central stencil for the interior datapoints and a first‐order forwards/backwards stencil for the first and last boundary values respectively (Fornberg 1988; LeVeque 2007, 3–11). The first‐order stencil for velocity is the derivative estimate at the current timestep using either the previous time‐step (backwards stencil) or next time‐step (forwards stencil). The second‐order central stencil is effectively the average of the forward and backwards first‐order stencil. Consequently, the boundary values have the greatest experimental uncertainty due to this first‐order Taylor series leading error term. These boundary values may be omitted but are acceptable to retain on a closed dataset for comparison's sake. With this velocity data and the individual bird masses, the instantaneous kinetic energy of the birds is known. Consequently, for each time value, any change in total energy (the sum of kinetic and potential) represents only kinematic work. From each incremental change in time frame to frame, the incremental change in work is then used to compute an instantaneous kinematic power output. The distance travelled over each frame is used with the work performed to calculate the instantaneous force output by the bird.

### 2.5 Statistical Analyses

All statistical analyses were performed in R (R Core Team 2021). To assess whether some of the variables are redundant and measure similar aspects of the flight performance, we conducted Spearman's rank correlations among our nine flight metrics, separately for the first and second set of flights, using the R package *corrr* (Kuhn et al. 2022). Spearman's rank correlations were used as some variables did not follow a normal distribution. We considered variables as highly correlated if the correlation coefficient was ≥ 0.8 and excluded redundant variables from further analyses such that only one of the highly correlated variables was kept (see Section 3 and Table A1 for details).

We tested whether our remaining flight metrics are meaningful and suitable for the detection of consistent individual differences in escape response with repeatability analyses of our remaining five variables across the two flights of each bird, using the *rptR* package (Stoffel et al. 2017). All repeatability analyses included bird ID as a random term. Repeatability values were considered significant if their 95% confidence intervals did not include zero. We first calculated repeatability using the full dataset (and models without any fixed terms). Given that there was variation in flightpath gradients (alpha) between paths, and that alpha was a significant predictor of some flight metrics (see Section 3), we were also interested in whether alpha would influence repeatability of those and other flight metrics. We assessed this in two different ways: first, we included average flight angle of each flight as a fixed term (adjusted repeatability), and second, we calculated repeatabilities after removing individuals with drastic differences in alpha between their two paths (Figure 2B). On the basis of examining the frequency distribution of flightpath gradient differences, we ran these models using flight tracks with less than a 20° difference that formed the bulk of the distribution (Figure 2B) (100 of 135 total individuals with data from two trials), as well as a more relaxed cutoff of 30° difference (117 of 135 total individuals with data from two trials); this let us include a larger number of birds, but there was more variation in flightpath gradients.

Finally, we investigated if the subspecies and their hybrids differed in their flight performance. If our results indicated the flight performance metrics differed between these groups, they may imply a selective difference between the subspecies and/or their hybrids. For these analyses we focused on the two flight metrics identified as repeatable (average force and maximum vertical distance over the flight; see Section 3). Given some group‐sex combinations were kept in aviaries, and some in cages, we could not analyse all data together in one analysis and could not make all comparisons without potential confounding sex or holding condition effects. Consequently, we ran three sets of linear mixed models per each flight metric (response variable, centred and scaled) via the *lmer* function of the *lme4* package (Bates et al. 2015), with residuals examined to check model assumptions and bird ID as a random term. Each set of models tested a different main prediction: First, we compared flight performances of *hecki* versus *acuticauda* males (all kept in cages); second, we compared flight performances of *acuticauda* versus hybrid females (all kept in aviaries) and finally, we compared flight performances of hybrid males versus hybrid females (all kept in aviaries). The first model tests for introgressive asymmetry for flight performance, i.e., if *hecki* displays escape flight performance superior to *acuticauda*. The second model examines the potential effect of mitodiscordance, i.e., if the *acuticauda* mitochondria interacting with the *hecki* Z chromosome (in the hybrid females) negatively impacts flight performance compared to the *acuticauda* mitochondria interacting with the *acuticauda* Z chromosome (in the *acuticauda* females). The third model compares if female hybrids perform worse than male hybrids due to the complete (in females) and partial (in males) incompatibility between mitochondria and Z chromosomes. As fixed terms we added body mass, average flight path gradient (both centred and scaled) and, depending on the model, either the respective group (*hecki*, *acuticauda* and/or hybrid; model 1: *hecki* vs. *acuticauda* males; model 2: hybrid vs. *acuticauda* females) or sex (model 3: male vs. female hybrids). We also ran these models with just the subset of data for which individuals had known tarsus length, which was used to calculate body condition and included as an additional fixed term. This allowed assessing if absolute or relative body mass was more important (if any) in explaining variation in the two flight metrics.

Non‐significant terms were removed in a stepwise manner, using likelihood ratio tests, until only statistically significant terms remained.

### 2.6 Ethics Statement

The work reported here was approved by the Macquarie University Animal Ethics Committee under ARA 2015/046 and 2020/025.

## 3 Results

The long‐tailed finches varied greatly in all flight parameters recorded across individuals (Table 1). For example, the total distance moved varied from 0.4 to 3 m. Some individuals flew most of this distance horizontally, others both vertically and horizontally, which is also seen in large variation in flight path gradients between trials. We calculated Spearman's rank correlations among our nine metrics and found that there was a moderate to high level of correlation among many of our metrics for both the first and second set of flights (Table A1). The highest correlations were observed between average velocity and total distance, followed by total distance and maximum horizontal distance and by average velocity and maximum horizontal distance (all *R*_s_ > 0.9). These findings are perhaps not surprising given that movements were followed for 0.625 s for all birds, therefore the maximum distance travelled in this time is similar to the average speed into the horizontal, if the vertical component is small relative to the horizontal component. Of these three redundant variables, only maximum horizontal distance was kept in for the repeatability analysis. Similarly, the maximum velocity and average power correlated highly with other variables (all *R*_s_ > 0.8) and were also removed from repeatability analysis.

**TABLE 1:** Descriptive statistics of the nine flight metrics calculated in this study in *P. acuticauda* subspecies and their hybrids (F1). Means [±1 SD] and ranges are shown separately for the first and second test series (trial).

Variable	Mean (SD)		Range	
	First trial	Second trial	First trial	Second trial
dTotal	1.93 (0.46)	1.51 (0.54)	0.79–3.02	0.39–2.78
dHMax	1.71 (0.45)	1.33 (0.48)	0.60–2.97	0.37–2.44
dVMax	0.82 (0.36)	0.61 (0.40)	0.10–1.79	0.00–1.63
vAvg	3.11 (0.73)	2.44 (0.87)	1.28–4.87	0.63–4.48
vMax	5.81 (1.44)	4.64 (1.50)	2.63–11.75	1.46–8.36
alphaAvg	26.02 (11.86)	23.69 (16.4)	−0.83 to 57.74	−49.42 to 58.34
alphaMax	43.21 (17.44)	45.61 (21.44)	14.44–89.75	−44.09 to 87.23
Pavg	0.57 (0.25)	0.39 (0.22)	0.16–1.75	−0.21 to 1.09
Favg	0.18 (0.05)	0.15 (0.06)	0.08–0.45	−0.10 to 0.29

Abbreviations: alphaAvg, flightpath gradient averaged for the flight [°]; alphaMax, maximum flightpath gradient for the flight [°]; dHMax, maximum horizontal distance [m]; dTotal, total distance travelled [m]; dVMax, maximum vertical distance [m]; Favg, average force [N]; Pavg, average power [W]; vAvg, average velocity [m/s]; vMax, maximum velocity [m/s].

For repeatability, only one out of five remaining flight parameters was significantly repeatable when considering all birds, which was the maximum vertical distance travelled (Table A2). Similarly, when flightpath gradient was fixed, maximum vertical distance was the only repeatable flight measure (Table A2). When we introduced cut‐offs to remove birds that flew at dramatically different flightpath gradients, two flight measures were significantly repeatable; repeatability was significant also for the average force in addition to the maximum vertical distance over the flight, regardless of whether we used a strict or relaxed threshold (< 20° and < 30° difference between first and second flight, respectively; Table A2). Intriguingly, despite the variation in flightpath gradient appearing to influence repeatability, including flightpath gradient as a fixed effect when calculating repeatability did not noticeably increase repeatability values (Table A2).

As average force and maximum vertical distance showed some evidence of repeatability, we focused our following analyses on them; both metrics were retained as response variables in subsequent mixed models since they were only moderately correlated (*R*_s_ < 0.7 for both first and second flight; Table A1).

**FIGURE 3:**
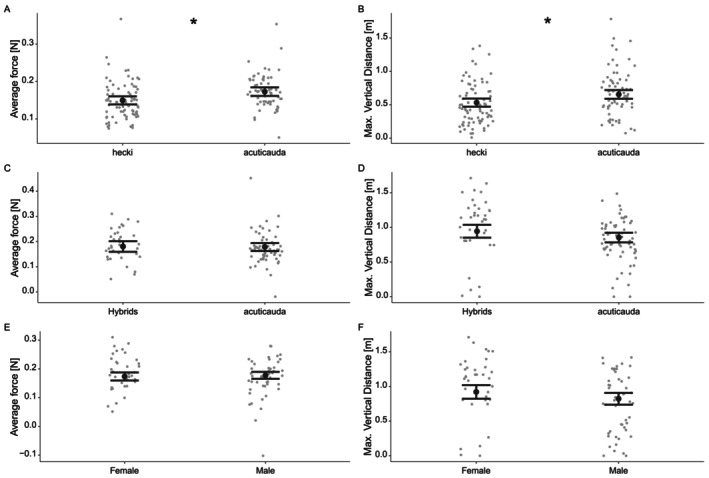
Flight parameters of different long‐tailed finch group‐sex comparisons for (A, B) male *hecki* versus male *acuticauda*, (C, D) female *acuticauda* versus female hybrids and (E, F) hybrid males versus hybrid females: Average force [N] and maximal vertical distance [m]. Grey points represent data of individual trials, the model estimates are in black indicating the mean and 95% confidence interval (CI) of the mean. Asterisks (*) indicate significantly different groups based on our mixed model accounting for flight angle, specifically that *hecki* males produced less force than *acuticauda* males and that *hecki* males achieved less vertical distance than *acuticauda* males.

Male *hecki* and male *acuticauda* individuals differed significantly in their average force and their maximum vertical distance travelled. *Hecki* males flew with lower average force and at lower height than *acuticauda* males (Figure 3A,B; Table A3). Flightpath gradient predicted average force and maximum vertical distance travelled, with higher average force and maximum vertical distance travelled for higher flightpath gradients, while there were no significant effects of body mass or body condition (Table A3).

Female *acuticauda* and female hybrids did not significantly differ in either the average force or the maximum vertical distance travelled (Figure 3C,D; Table A3). With increasing flightpath gradient, both the average force and maximum vertical distance travelled increased across those females, and maximum vertical distance also increased with body mass (Table A3). Body condition did not predict either flight metric.

Finally, there were no significant differences in the average force and the maximum vertical distance travelled between male and female hybrids (Figure 3E,F; Table A3). As in previous comparisons, trials with larger flightpath gradients had higher average force and maximum vertical distance travelled; body mass and body conditions were not significant predictors of either average force or maximum vertical distance in hybrid birds.

## 4 Discussion

Here, we showed that individual long‐tailed finches differ consistently in aspects of their escape flight response, namely average force and maximal vertical distance. We also detected a significant difference in the escape flight of the two subspecies, with the flights of *hecki* males having lower average force values and lower maximum vertical distances than those of *acuticauda* males. If average force is considered a representative measure of flight performance, and if the asymmetric introgression observed in wild long‐tailed finches is a predictor of superior *hecki* fitness, it is surprising that *hecki* achieved significantly reduced average force compared to *acuticauda*. The introgressive asymmetry of mito‐types observed in wild long‐tailed finches (Lopez et al. 2021) would have predicted the opposite trend, whereby *hecki* achieves significantly greater flight performance. These results may indicate that selection upon flight performance is not a primary driver of any net selective differences between the two subspecies and does not account for the introgressive asymmetry observed in the wild (Hooper et al. 2019). It is plausible that other traits are under selection in wild populations or that intraspecific differences in dispersal behaviours or genetic drift may play a role. It is also worth noting that we only measured voluntary flight take‐off behaviour and hence the number and kind of flight metrics we recorded might not capture important measures of flight performance in other contexts. For example, perhaps endurance flight might better reflect variation in mitochondrial performance than take‐off performance (Ton et al. 2025). Take‐off performance may instead be linked to short‐term escape/risk‐taking behaviour, where perhaps *acuticauda* individuals might invest into quickly escaping potential predation risk, whereas *hecki* may take higher risks, possibly explaining differences between the two subspecies. This potential explanation would require further testing, but in line with this interpretation, fire salamander, *Salamandra salamandra* , individuals of the introgressed mito‐type were more cautious compared to individuals of the introgressing mito‐type (Chiocchio et al. 2024).

Evolutionary theory would also predict that the F1 hybrids may display poorer flight performance compared to the parental subspecies, due to the effects of outbreeding depression on the phenotype, or the mismatch between the mitochondrial genes and the nuclear genes that interact with them to ensure optimal mitochondrial function (Hill 2017; Koch et al. 2021). It is surprising that hybrid females did not have reduced flying capacity compared to *acuticauda* females as, in the wild, hybrids appear to have reduced fitness (Hooper et al. 2019; Lopez et al. 2021), however, it could be that selection acts on later hybrid generations for which there has been more opportunity for genetic admixture (Johannesen et al. 2006; Wiley et al. 2009). We had expected to have found a difference in the flight take‐off performance of F1 hybrid females in contrast to F1 hybrid males because the former will have a mismatch between their mitochondrial genes and mitonuclear genes (Lopez et al. 2021; Pacheco‐Fuentes et al. 2025), while the males will not. While recent work in other contexts has identified functional differences in aspects of mitochondrial respiration in the context of mitonuclear incompatibility between these two subspecies (McDiarmid et al. 2024; Pacheco‐Fuentes et al. 2025), our failure to find any difference here between the male and female F1 hybrids suggests that this take‐off assay is not capturing variation in mitochondrial respiration performance in these birds. One possible reason for that may be that the first seconds of flight, as measured here, are largely anaerobic, and perhaps not determined by differences in mitochondrial respiration. Indeed, recent work in another passerine, the house sparrow, *Passer domesticus* , suggests that only sustained exercise in birds is determined by variation in mitochondrial respiration, and not take‐off flight (Ton et al. 2025).

We found little clear support for an influence of body mass or condition on flight performance. Body mass only weakly positively predicted maximal vertical distance, but not average force, and only in one of the comparisons. Similarly, there was no evidence of body condition correlating with either flight metric. This may appear surprising but it should also be noted that in the literature, there is some skepticism as to whether mass or tarsus length metrics are true reflections of body condition in captive birds with access to ad libitum food (Birkhead et al. 1998).

The methodology employed in our study holds promise to expand research on the ecology and evolution of avian flight (see also Jackson et al. 2016a; Swaddle et al. 2020; Thady et al. 2022); these methods may be repeated to obtain flight metrics to analyse bird flight without elaborate laboratory set‐ups and in field conditions. However, there are a couple of refinements that may be worth considering. First, the flightpath gradient strongly influenced flight metric values, and it would be useful to modify the method to better standardise the flight path, and thus flight angle, of the birds. For example, introducing some constraints for where individuals can fly when first released. It is unknown whether flight angle indicates inherent flying ability/power or simply a behavioural choice the birds made. While we did demonstrate a good level of repeatability in two of our performance measures, it is unknown to which extent this was driven by energetic differences between birds, or some behavioural or motivational difference, and this would be worth exploring in future studies.

In summary, our results do not support our prediction, based on the divergence in mito‐types between the subspecies, and asymmetric introgression of mitochondrial genes (Lopez et al. 2021), that *hecki* would display significantly greater flight take‐off performance compared to *acuticauda*. Furthermore, we failed to find any evidence to support the idea that mitonuclear discordance would more adversely affect the flight performance of hybrids, and particularly females. Nevertheless, we have demonstrated significant inter‐individual and inter‐subspecific differences in some key components of take‐off flight, suggesting that they can diverge relatively quickly and potentially play an important role in natural selection in birds.

Our study provides encouraging signs that the methodology can measure biologically important variation in bird ‘escape flights’ across individuals or populations in other species and evolutionary contexts. Future studies should investigate additional traits that may be under selection in wild long‐tailed finches and that likely contribute to the observed asymmetric introgression between these two subspecies.

## Author Contributions

**Samuel Ashby:** conceptualization (equal), data curation (lead), formal analysis (equal), investigation (lead), methodology (lead), visualization (equal), writing – original draft (lead), writing – review and editing (supporting). **Callum S. McDiarmid:** conceptualization (equal), data curation (supporting), formal analysis (equal), investigation (supporting), methodology (lead), supervision (supporting), validation (equal), writing – original draft (supporting), writing – review and editing (supporting). **Dylan Dooner:** formal analysis (supporting), visualization (supporting), writing – original draft (supporting), writing – review and editing (supporting). **Simon C. Griffith:** conceptualization (lead), funding acquisition (lead), methodology (supporting), project administration (equal), resources (lead), supervision (supporting), writing – review and editing (equal). **Wiebke Schuett:** conceptualization (equal), formal analysis (equal), methodology (supporting), project administration (equal), supervision (lead), visualization (equal), writing – review and editing (equal).

## Funding

This work was supported by Australian Research Council (DP180101783).

## Conflicts of Interest

The authors declare no conflicts of interest.

## Data Availability

The data that support the findings of this study are available on *Figshare*: https://doi.org/10.25377/sussex.31765711 (Ashby et al. 2026).

## Appendix A

**TABLE A1:** Spearman's rank correlation coefficients between the nine‐flight metrics calculated in this study.

	*dTotal*	dHMax	dVMax	*vAvg*	*vMax*	alphaAvg	alphaMax	*Pavg*	Favg
*dTotal*	NA	**0.924**	0.422	**0.998**	0.774	−0.027	−0.229	0.711	0.250
dHMax	**0.958**	NA	0.102	**0.924**	0.698	−0.318	−0.419	0.526	0.051
dVMax	0.656	0.471	NA	0.420	0.403	0.766	0.393	0.646	0.588
*vAvg*	**0.998**	**0.957**	0.653	NA	0.775	−0.029	−0.227	0.712	0.251
*vMax*	0.856	0.860	0.527	0.854	NA	0.054	0.127	0.895	0.633
alphaAvg	0.225	0.019	0.793	0.220	0.170	NA	0.719	0.328	0.521
alphaMax	−0.194	−0.288	0.220	−0.206	−0.005	0.605	NA	0.285	0.599
*Pavg*	0.859	0.780	0.795	0.856	0.882	0.469	0.132	NA	0.818
Favg	0.409	0.308	0.639	0.403	0.620	0.559	0.463	0.785	NA

*Note:* Values in top right triangle are correlation coefficients for first test series (shaded in grey), values in bottom left triangle for second test series. Correlation coefficients over 0.8 were considered high, are marked in bold and variables involved considered as redundant; variables taken out as a consequence are marked in italics.

Abbreviations: alphaAvg, flightpath gradient averaged for the flight [°]; alphaMax, maximum flightpath gradient for the flight [°]; dHMax, maximum horizontal distance [m]; dTotal, total distance travelled [m]; dVMax, maximum vertical distance [m]; Favg, average force [N]; Pavg, average power [W]; vAvg, average velocity [m/s]; vMax, maximum velocity [m/s].

**TABLE A2:** Repeatability (R) of flight metrics.

Dataset	Flightpath gradient fixed term	*N*_observations_ (*N*_individuals_)	Variable	*R*	95% CI	
Full	No	291 (158)	dHMax	0	0	0.155
			**dVMax**	**0.267**	**0.104**	**0.420**
			alphaAvg	0.114	0	0.273
			alphaMax	0.143	0	0.307
			Favg	0.106	0	0.271
	Yes		dHMax	0	0	0.167
			**dVMax**	**0.284**	**0.117**	**0.431**
			Favg	0.103	0	0.262
Gradient difference < 20°	No	200 (100)	dHMax	0	0	0.203
			**dVMax**	**0.516**	**0.355**	**0.644**
			**Favg**	**0.264**	**0.070**	**0.44**
Gradient difference < 30°	No	234 (117)	dHMax	0.003	0	0.196
			**dVMax**	**0.445**	**0.280**	**0.580**
			**Favg**	**0.286**	**0.101**	**0.448**

*Note:* Flightpath gradient was included as a fixed effect for some models, or datasets were filtered to remove individuals with large differences in flightpath gradient between their two flights. Values in bold indicate low to moderate repeatability.

Abbreviations: alphaAvg, flightpath gradient averaged for the flight [°]; alphaMax, maximum flightpath gradient for the flight [°]; dHMax, maximum horizontal distance [m]; dVMax, maximum vertical distance [m]; Favg, average force [N].

**TABLE A3:** Linear mixed model output testing predictors of average force (Favg [N]) and maximum vertical distance (dVMax [m]).

Data	*N* (*N*_ID_)	Response	Random term	Variance	Fixed term	Coef.^b^	*χ* ^2^	*p*
M *hecki*	145 (79)	Favg^a^	Bird ID	0.07	Intercept	−0.28		
M *acuticauda*			(Residual)	0.53	Group [*acuticauda*]	0.40	8.49	**0.004**
					Mass^a^	(0.03)	0.22	0.640
					alphaAvg^a^	0.33	15.85	**< 0.0001**
		dVMax^a^	Bird ID	0.03	Intercept	−0.41		
			(Residual)	0.41	Group [*acuticauda*]	0.31	7.37	**0.007**
					Mass^a^	(< −0.01)	< 0.01	0.939
					alphaAvg^a^	0.64	68.56	**< 0.0001**
F *acuticauda*	98 (54)	Favg^a^	Bird ID	0.16	Intercept	0.11		
F hybrids			(Residual)	0.81	Group [*acuticauda*]	(< −0.01)	< 0.01	0.965
					Mass^a^	(0.08)	0.20	0.657
					alphaAvg^a^	0.44	20.32	**< 0.0001**
		dVMax^a^	Bird ID	0.16	Intercept	0.43		
			(Residual)	0.16	Group [*acuticauda*]	(−0.14)	0.84	0.361
					Mass^a^	0.25	4.94	**0.026**
					alphaAvg^a^	0.67	107.67	**< 0.0001**
M hybrids	84 (44)	Favg^a^	Bird ID	< 0.01	Intercept [f]	0.04		
F hybrids			(Residual)	0.50	Sex [m]	(0.07)	0.18	0.670
					Mass^a^	(−0.05)	0.35	0.557
					alphaAvg^a^	0.72	79.95	**< 0.0001**
		dVMax^a^	Bird ID	0.11	Intercept [f]	0.27		
			(Residual)	0.34	Sex [m]	(−0.25)	2.38	0.123
					Mass^a^	(0.01)	0.16	0.899
					alphaAvg^a^	0.76	94.65	**< 0.0001**
M *hecki*	93 (49)	Favg^a^	Bird ID	0.09	Intercept	−0.22		
M *acuticauda*			(Residual)	0.37	Group [*acuticauda*]	0.41	6.24	**0.012**
					Mass^a^	(−0.06)	0.11	0.737
					alphaAvg^a^	0.29	10.45	**0.001**
					Condition^a^	(0.14)	3.59	0.060
		dVMax^a^	Bird ID	0.03	Intercept	−0.38		
			(Residual)	0.45	Group [*acuticauda*]	0.41	6.89	**0.009**
					Mass^a^	(0.14)	3.09	0.079
					alphaAvg^a^	0.51	28.07	**< 0.0001**
					Condition^a^	(−0.16)	1.28	0.257
F *acuticauda*	45 (24)	Favg^a^	Bird ID	< 0.01	Intercept	0.05		
F hybrids			(Residual)	0.62	Group [*acuticauda*]	(−0.13)	0.17	0.677
					Mass^a^	(−0.18)	0.52	0.471
					alphaAvg^a^	0.61	21.96	**< 0.0001**
					Condition^a^	(0.05)	0.11	0.742
		dVMax^a^	Bird ID	0.22	Intercept	0.37		
			(Residual)	0.27	Group [*acuticauda*]	(−0.26)	0.77	0.381
					Mass^a^	(0.24)	2.10	0.147
					alphaAvg^a^	0.69	40.94	**< 0.0001**
					Condition^a^	(−0.13)	0.29	0.590
M hybrids	84 (44)	Favg^a^	Bird ID	< 0.01	Intercept [f]	0.04		
F hybrids			(Residual)	0.50	Sex [m]	(0.10)	0.36	0.547
					Mass^a^	(0.03)	0.02	0.883
					alphaAvg^a^	0.72	79.95	**< 0.0001**
					Condition^a^	(−0.05)	0.40	0.528
		dVMax^a^	Bird ID	0.11	Intercept [f]	0.27		
			(Residual)	0.34	Sex [m]	(−0.25)	2.38	0.123
					Mass^a^	(0.18)	1.11	0.292
					alphaAvg^a^	0.76	95.32	**< 0.0001**
					Condition^a^	(−0.04)	0.22	0.641

*Note:* Models were repeated for those individuals with tarsus measures that were used to calculate body condition and added as additional fixed term, lower set of models. Random term was bird ID throughout. Significant *p*‐values in bold. Coefficients (Coef.) of non‐significant terms are from the time a term was dropped from the model and are not comparable to coefficients from minimal adequate model.

Abbreviations: alphaAvg, flightpath gradient averaged for the flight [°]; Df, degrees of freedom; dVMax, maximum vertical distance [m]; F, female; Favg, average force [N]; M, male; *N*, number of trials; *N*_ID_, number of individuals.

^a^ Data centred and scaled.

^b^ Model with close to zero variance explained by ‘bird ID’ and/or convergence issue—qualitatively the same results obtained using GLM without random term.
